# Supercapacitive microbial desalination cells: New class of power generating devices for reduction of salinity content

**DOI:** 10.1016/j.apenergy.2017.10.056

**Published:** 2017-12-15

**Authors:** Carlo Santoro, Fernando Benito Abad, Alexey Serov, Mounika Kodali, Kerry J. Howe, Francesca Soavi, Plamen Atanassov

**Affiliations:** a Department of Chemical and Biological Engineering, Center for Micro-Engineered Materials (CMEM), University of New Mexico, Albuquerque, NM 87131, USA; b Department of Civil Engineering, Center for Water and the Environment, University of New Mexico, MSC01 1070, Albuquerque, NM 87131, USA; c Department of Chemistry “Giacomo Ciamician“, Alma Mater Studiorum – Universita’ di Bologna, Via Selmi 2, 40126 Bologna, Italy

**Keywords:** AC, activated carbon, AdE, additional electrode, AEM, anion exchange membrane, BES, bioelectrochemical system, C_anode_, anode capacitance, CB, carbon black, C_cathode_, cathode capacitance, C_cell_, cell capacitance, CDI, capacitive deionization, Cell ESR, equivalent series resistance of the cell, CEM, cation exchange membrane, DC, desalination chamber, DI, deionized water, EDLC, electrochemical double layer capacitor, E_pulse_, energy obtained by the pulse, Fe-AAPyr, iron aminoantypirine, GLV, galvanostatic discharges, i_pulse_, , current pulses, KCl, potassium chloride, KPB, potassium phosphate buffer, MDC, membrane capacitive deionization, MDC, microbial desalination cell, MFC, microbial fuel cell, MFC, microbial fuel cell, NaCl, sodium chloride, NaOAc, sodium acetate, OCV, open circuit voltage, ORR, oxygen reduction reaction, PGM-free, platinum group metals-free, P_max_, maximum power, P_pulse_, power obtained by the pulse, PTFE, polytetrafluoroethylene, R_A_, anodic anode ohmic resistance, R_C_, cathodeic ohmic resistance, RO, reverse osmosis, SC-MDC-AdE, supercapacitive microbial desalination cell with additional electrode, SC-MDC, supercapacitive microbial desalination cell, SC-MFC, supercapacitive microbial fuel cell, SC, solution conductivity, SHE, standard hydrogen electrode, t_pulse_, time of the pulse, t_rest_, rest time, V^−^, _oc_, anode potentials in open circuit, V^+^, _oc_, cathode potential in open circuit, V_max_, _OC_, original maximum voltage in open circuit condition, V_max_, practical voltage, ΔV_capacitive_, difference between V_max_ and V_final_ (at the end of t_pulse_), voltage capacitive decrease drop, ΔV_ohmic_, _cathode_, cathode ohmic drop, ΔV_ohmic_, difference between V_max,OC_ and V_max_, ohmic drop, Supercapacitive Microbial Desalination Cell (SC-MDC), Additional Electrode (AdE), Power/current pulses, High power generation, Transport phenomena

## Abstract

In this work, the electrodes of a microbial desalination cell (MDC) are investigated as the positive and negative electrodes of an internal supercapacitor. The resulting system has been named a supercapacitive microbial desalination cell (SC-MDC). The electrodes are self-polarized by the red-ox reactions and therefore the anode acts as a negative electrode and the cathode as a positive electrode of the internal supercapacitor. In order to overcome cathodic losses, an additional capacitive electrode (AdE) was added and short-circuited with the SC-MDC cathode (SC-MDC-AdE). A total of 7600 discharge/self-recharge cycles (equivalent to 44 h of operation) of SC-MDC-AdE with a desalination chamber filled with an aqueous solution of 30 g L^−1^ NaCl are reported. The same reactor system was operated with real seawater collected from Pacific Ocean for 88 h (15,100 cycles). Maximum power generated was 1.63 ± 0.04 W m^−2^ for SC-MDC and 3.01 ± 0.01 W m^−2^ for SC-MDC-AdE. Solution conductivity in the desalination reactor decreased by ∼50% after 23 h and by more than 60% after 44 h. There was no observable change in the pH during cell operation. Power/current pulses were generated without an external power supply.

## Highlights

• The concept of supercapacitive microbial desalination cell is here presented.
• The device is able to degrade organics, desalinate and generate power simultaneously.
• An additional electrode overcomes cathode ohmic losses and boost up power output.
• Maximum power achieved was 3.0 W m^−2^ (2.1 mW).
• 7600 discharge/self-recharge cycles were demonstrated over 44 h.

## Graphical abstract

Supercapacitive Microbial Desalination Cell is here presented with unprecedented performances. Anode and cathode act as negative and positive electrode of an internal supercapacitor that is discharged and self-recharged. Maximum power of 3 W m^−2^ is recorded.

**Figure:**
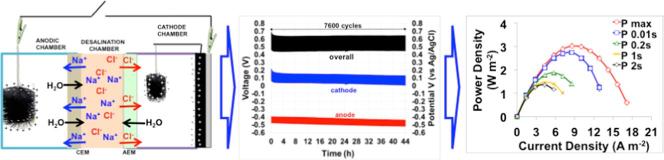

## 1 Introduction

The constant increase in living standards has increased utilization of natural resources, including a depletion of water resources and a dramatic decrease in the water quality in natural water sources. Water scarcity, water quality, and related sanitation issues are tremendous problems in poor and developing countries. High energy consumption is also important for water treatment. Nowadays, the majority of the energy utilized is derived from fossil fuels rather than from renewable sources. Water and energy related subjects are the two most problematic challenges that humans have to face and solve. Therefore, sustainable and alternative solutions need to be investigated.

The majority of the available water on Earth (over 96.5%) is salty water coming from the oceans that count for over 71% of the planet surface [1], [2], [3], [4], [5]. It seems natural to recover drinkable water from salty water. Several desalination technologies have been successfully explored over time. Despite high efficiency reached in desalinating, the main factors that limit the large-scale application worldwide seems to be the elevated cost and energy consumption [6], [7], [8]. The main existing technologies used are based on utilization of either heat (distillation) or membranes (reverse osmosis or electrodialysis). Presently, distillation produces roughly 60% of all drinking water obtained by desalination but the main problem of this technology is that the desalination plant has to be located in a proximity of a power plant and use its waste heat [9], [10], [11], [12].

The second method is based on utilization of membranes, such as reverse osmosis [13], [14], [15], [16] and nanofiltration [17], [18], [19], [20] for large-scale desalination of water. These processes are driven by the application of an external pressure to overcome the natural osmotic pressure and forcibly push the water through the membrane. The main difference between reverse osmosis and nanofiltration is that the first one theoretically is able to eliminate all the ions while the second one primarily removes divalent ions. Thus, nanofiltration is not suitable for seawater desalination since that mainly consists of monovalent ions. Reverse osmosis is very expensive due to the membrane cost and the utilization of a large amount of energy. It is not surprising that reverse osmosis is mainly used in highly developed countries and in countries with large availability of inexpensive sources of energy derived from fossil fuels.

Another technology used is electrodialysis, in which positive and negative electrodes create an electric field that separates ions by migration towards opposite charged electrodes [21], [22], [23], [24]. Under externally applied potentials, the anode is positively charged, cathode is negatively charged, and ion flux is controlled by anion exchange membranes facing the anode electrode (positively charged) and cation exchange membranes facing the cathode electrode (negatively charged). The charged electrodes attract counter-ions from the central flow through specific membranes. This method also requires a considerable amount of energy.

Capacitive deionization (CDI) is another promising technology under consideration [25], [26], [27], [28]. It is based on utilization of high surface area carbon materials at two electrodes. A potential difference is applied to charge positive (anode) and negative (cathode) porous electrodes [25], [26], [27], [28]. CDI is based on two consequent processes of adsorption and desorption in which ions are first separated from the salty water and therefore water is desalinated. In the adsorption process, electrical double layers (EDLs) are formed on both charged electrodes through the attraction of ions that are separated from the water. The solution between the electrodes is replaced and the electrodes are then discharged to null voltage, energy is delivered, and ions are released into the solution (which becomes a waste stream) [25], [26], [27], [28]. A technology slightly different from CDI is achieved with the addition of membranes, known as membrane capacitive deionization (MCD) in which anion-selective membrane is inserted on the positive electrode and a cation exchange membrane is used on the negative electrode [29], [30], [31], [32], [33]. Compared to CDI, MDI is able to operate with lower energy consumption and better salt separation. However, the membranes significantly increase the overall costs of desalination system. In both cases, the discharges processes take place with a potential generated that is quite low (not greater than 200–300 mV) and consequently energy recovered cannot be used for any practical application.

New technologies for desalinating of salty water or reducing the salt content within a water stream have been recently introduced with promising results [34], [35], [36], [37], [38]. Particularly, microbial desalination cells (MDC) have captured the interest of the scientific community. The most studied bioelectrochemical systems (BES) are microbial fuel cells (MFC) [34], which are electrochemical devices in which electroactive bacteria are the anodic catalysts and able to oxidize pollutants and/or transform nutrients [39], [40], [41], [42], [43], [44]. An MDC is a BES device derived from a microbial fuel cell in which anode and cathode compartments are further divided by ionic selective membranes (anion and cation exchange membrane) [35], [45], [46]. While current is generated due to the organic degradation at the anode and the oxygen reduction reaction (ORR) at the cathode, ions move through the exchange membranes, mainly due to osmosis and diffusion [47], [48], [49], [50], [51]. Interestingly, the open circuit voltage (OCV) is similar to the voltage in MFCs but total power and current generated are lower due to losses associated with addition of membranes [52]. The result is a tri-generative device that simultaneously treats wastewater by degrading organic pollutants, produces electricity, and decreases the salt content in the desalination chamber [47], [48], [49], [50]. Several examples of MDCs have been shown in literature [53], [54], [55], [56], [57], [58], [59], [60], [61], [62], [63].

Several challenges have to be overcome for MDC technology to be viable; for example, the ion flux rates are low and the extent of desalination is significantly lower than existing desalination technologies. Another problem is related with the low power production from MDC that is 2–3 times lower than MFCs [47], [48], [49]. Moreover, electrode materials (anode and cathode) have to be tested in long term operations and costs have to be significantly reduced to be competitive with other desalination technologies [47], [48], [49]. Anode materials need to possess mechanical strength, resistance to bio-corrosion, and high electrical conductivity. Concerning the cathode materials, the cost has to be decreased and platinum cathode catalysts have to be replaced with more affordable and higher performing carbonaceous high surface area catalysts [34], [47], [48], [49], [50] or platinum group metals-free (PGM-free) catalysts [34], [47], [48], [49], [50]. In parallel, membrane costs have to be decreased with substantial increase in membrane durability. At the moment, membranes are a significant contributor to the cost of the entire MDC system. Membrane fouling and biofouling seem also to affect negatively the ion exchange rates over time and decrease the performance [47], [48], [49].

In this study, we combine the advantages of MDC and CDI within a novel system which we call a supercapacitive Microbial Desalination Cell (SC-MDC), presented here for the first time, in order to increase the power/current produced. The supercapacitive features of the MDC electrodes are used as an internal supercapacitor. The operation of SC-MDC in pulsed and intermittent modes over 44 h were reported. Power/current pulses are generated along with the decrease of salt content and organics. The electrochemical response of the SC-MDC is shown as well as solution conductivity and pH of the solutions monitored over time. An additional capacitive electrode (AdE) was used to overcome cathode ohmic losses and achieve higher power output. In order to decrease the overall costs, particular attention was devoted to the cathode and AdE materials that were strictly fabricated without the utilization of platinum but only with high surface area activated carbon and PGM-free catalysts. Finally, the same tests were conducted on SC-MDC and SC-MDC-AdE utilizing real seawater into the desalination chamber.

## 2 Experimental section

### 2.1 Configuration and working conditions

**Fig. 1:**
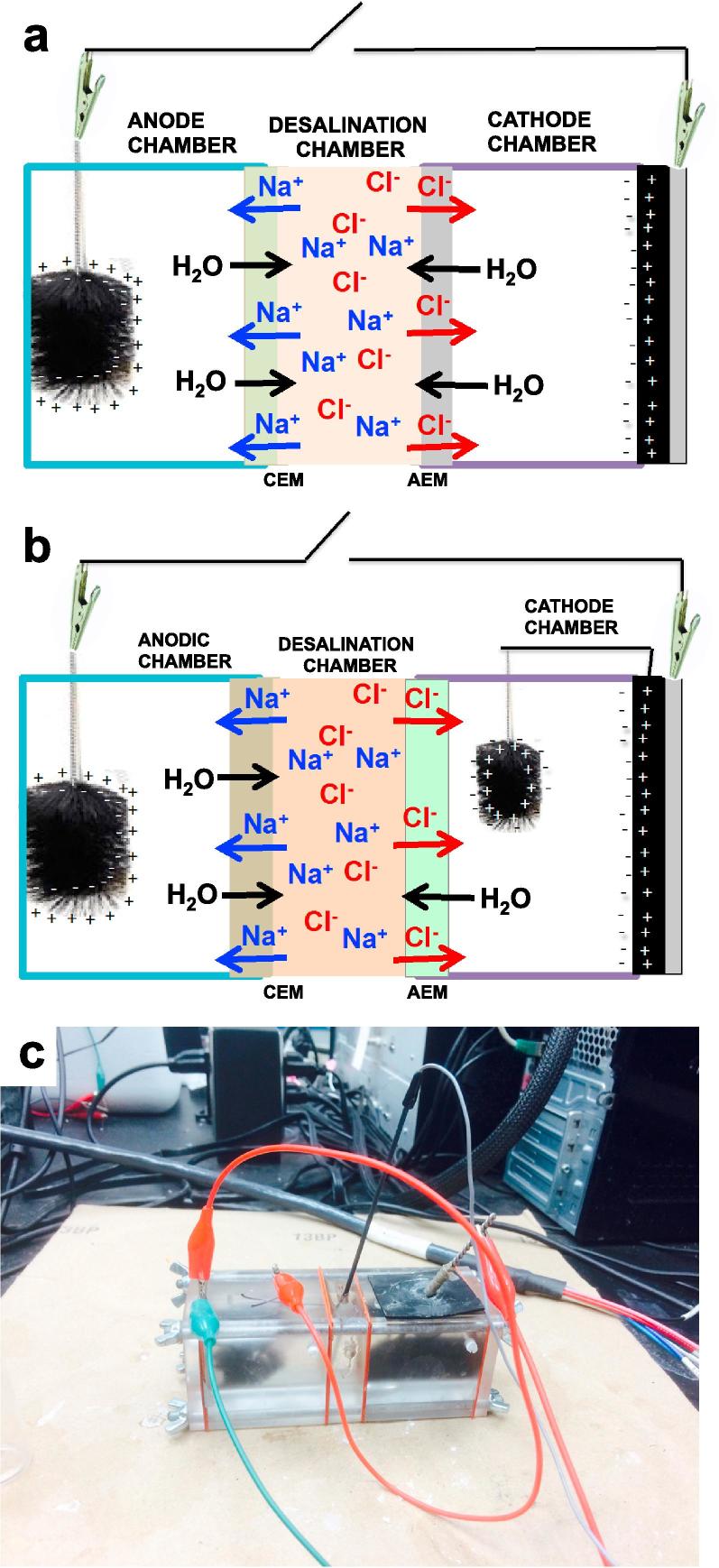
Schematic representation of: (a) supercapacitive MDC, (b) supercapacitive MDC with additional electrode (AdE); and (c) image of the working supercapacitive MDC with the AdE.

The SC-MDC (Fig. 1a) and SC-MDC-AdE (Fig. 1b) consisted of three compartments physically separated by polymeric ion-exchange membranes. A photograph of the working SC-MDC-AdE system is shown on Fig. 1c. The middle chamber of the system was the water desalination chamber (denoted as DC) and had a useful load volume of 11 mL. The anode chamber (volume of 35 mL) was separated from the DC by a cation exchange membrane (CEM, CSO, 100 μm, AGC Engineering CO., LTD, Japan). On the opposite side, the cathode chamber (volume of 35 mL) was separated from the DC by an anion exchange membrane (AEM, Fumapem FAA-3-50 non-reinforced, 50 μm, Fumatech GmbH, Germany). On one side of the cathode chamber, the air-breathing cathode was screwed to the plastic support, exposing one face to the cathode solution and one face directly to air. The anode chamber was filled with a solution composed of 50% by volume of 50 mM potassium phosphate buffer (KPB) and 50% by volume of activated sludge (Southside Wastewater Reclamation Plant, Albuquerque, NM, USA) with the addition of 3 g L^−1^ sodium acetate (NaOAc) as fuel for the electroactive bacteria. The cathode chamber was filled with a solution composed of 23 mM KPB. For different experiments, the desalination cell was filled with two different solutions separately: (i) a 30 g L^−1^ NaCl solution in DI water (SC of 48.4 ± 1.8 mS cm^−1^) in order to simulate seawater and (ii) real seawater collected from the Pacific Ocean in Solana Beach, CA (USA). The solution conductivity was measured using a conductivity meter (Orion Star A112, Thermo Scientific, USA). The anode chamber solution had an average initial conductivity of 8.5 ± 1.0 mS cm^−1^. The initial conductivity of the NaCl solution was 48.4 ± 1.8 mS cm^−1^ and that of the Pacific Ocean seawater was 51.9 ± 0.5 mS cm^−1^; the cathode chamber solution had an initial conductivity of 5.4 ± 0.9 mS cm^−1^.

The pH was measured using a Benchmeter OMEGA PHB-600 R (Norwalk, CT, USA) pH meter. COD was measured at the beginning and at the end of the experiments using standard HACH vials (Colorado, USA) and following the standard measurement method (HACH Method 8000).

### 2.2 Materials

The cation and anion exchange membranes and cathode each had an exposed geometric area of 7 cm^2^. The anode was a cylindrical carbon brush (3 cm diameter and 3 cm height, Millirose) with a projected area of 9 cm^2^ inserted into the anode chamber (Fig. 1). An electroactive biofilm was grown on the anode in separate MFCs and therefore at the beginning of the experiments, the anode was pre-colonized of electroactive bacteria and working well. As a result, the start up time for biofilm attachment and development was minimized. The cathode was an air-breathing gas diffusional electrode. The cathode pellet was composed of a mixture of activated carbon (AC, 70 wt% Norit SX Plus), carbon black (10 wt%, Alfa Caesar) and PTFE (20 wt%, 60% solution Sigma Aldrich) that was grinded in a blender for 5 min [64]. The obtained mixture was then further mixed with Fe-AAPyr used as oxygen reduction catalyst and then inserted in a pellet die and pressed at 2 mT for 5 min. Fe-AAPyr was previously identified as exceptional catalyst for oxygen reduction reaction in neutral media [65], [66], [67]. The AC+CB+PTFE loading was 40 mg cm^−2^ while Fe-AAPyr loading was 2 mg cm^−2^. The additional electrode (AdE) was a carbon brush (2 cm diameter and 2 cm height, Millirose) coated with activated carbon (AC) to increase the capacitive response.

### 2.3 Methods

Electrochemical measurements on the SC-MDC and SC-MDC-AdE were carried out using a BioLogic SP-50 potentiostat. SC-MDC featured a three-electrode setup with an Ag/AgCl (3M KCl, potential shift of +210 mV vs. SHE) reference electrode in the desalination chamber, anode as counter electrode and cathode as working electrode. In order to minimize the cathodic losses, the AdE was short-circuited with the cathode and the cell configuration was then called SC-MDC-AdE (Fig. 1). This methodology was previously presented [68], [69], [70], [71], [72]. In previously reported literature, the supercapacitive properties of the electrodes were tested in MFCs [68], [69], [70], [71], [72], whereas in this current work the same features were exploited and utilized in operating MDCs. Here, we demonstrate that these features not only increase the power/current output but also enhance the reduction in salt content.

Galvanostatic pulses at currents (i_pulse_) of 2 mA for 1 s (t_pulse_) followed by a rest period (t_rest_) of 20 s were repeated over 44 h with the DC filled with 30 g L^−1^ NaCl and over 88 h with the DC filled with real seawater. The operations were interrupted only during the sampling time. Solution conductivity and pH were measured over the duration of the experiments.

Galvanostatic discharge (GLV) curves were measured at various discharge currents (i_pulse_) while the anode and cathode potentials were monitored separately. After each pulse, the SC-MDC or SC-MDC-AdE was set in rest conditions until the original maximum voltage (V_max,OC_) was restored and consequently the internal SC-MDC or SC-MDC-AdE was self-recharged. At the beginning of the GLV pulse, the cell voltage decreased from V_max,OC_ to a practical voltage (V_max_) and this is due to the ohmic losses of the cell. The difference between V_max,OC_ and V_max_ (ΔV_ohmic_) depends on the equivalent series resistance (cell ESR) of the cell and includes the ohmic contributions of the electrolyte and of the electrodes. The calculation of cell ESR is shown in Eq. (1):

(1) $$\mathrm{cell}\;\mathrm{ESR}=\frac{\Delta V_{\mathrm{ohmic}\text{,}\mathrm{cell}}}{i_{\mathrm{pulse}}}$$

The separate electrode profiles during the GLV discharges were used to estimate each electrode resistance. The reference electrode was placed in the middle of the cell (in the DC) so that the ohmic losses of each electrode divided by the i_pulse_ give an approximation of the anodic (R_A_, Eq. (2)) and cathodic (R_C_, Eq. (3)) ohmic resistances.

(2) $$R_{A}=\frac{\Delta V_{\mathrm{ohmic}\text{,}\mathrm{anode}}}{i_{\mathrm{pulse}}}$$

(3) $$R_{C}=\frac{\Delta V_{\mathrm{ohmic}\text{,}\mathrm{cathode}}}{i_{\mathrm{pulse}}}$$

After the initial ohmic drop, the cell voltage decreased linearly over time (ΔV_capacitive_). The slope of the discharge voltage over time (dV/dt) is inversely related to the capacitance of the cell. Capacitance (C) was calculated the using Eq. (4):

(4) $$C_{\mathrm{cell}}=\frac{i}{\frac{\mathrm{dV}}{\mathrm{dt}}}$$

Anode (C_anode_) and cathode (C_cathode_) capacitances were similarly calculated (see Eq. (4)) but considering the slopes of the correspondent electrode potential profiles over time.

The maximum power output (P_max_) for each SC-MDC was obtained by multiplying V_max_ by i_pulse_. P_max_ value is the power that can is delivered by the device at the beginning of the pulse (after the ohmic drop).

(5) $$P_{\max}=V_{\max}\times i_{\mathrm{pulse}}$$

The voltage changes during the discharge of SC-MDC mainly due to the capacitive response of the cell and thus the actual power of the pulse (P_pulse_) is less than P_max_. P_pulse_ is calculated as the ratio between the energy (E_pulse_) delivered during the pulse and the duration time of the pulse (t_pulse_). E_pulse_ is calculated by the integration of the discharge curve over time according to the equation:

(6) $$E_{\mathrm{pulse}}=i\int_{0}^{t}V\;\mathrm{dt}$$

(7) $$P_{\mathrm{pulse}}=\frac{E_{\mathrm{pulse}}}{t_{\mathrm{pulse}}}$$

## 3 Principle of a SuperCapacitive Microbial Desalination Cell (SC-MDC)

**Fig. 2:**
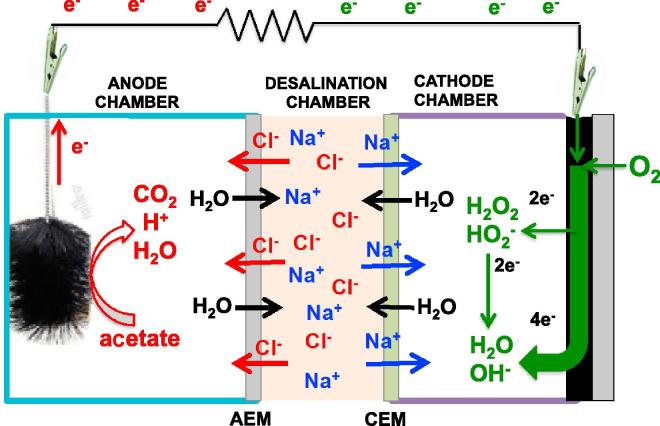
Schematic representation of a microbial desalination cell (MDC).

In this section, we describe the operating principle of the supercapacitive MDC (SC-MDC). In a working MDC, oxidation of organics takes place at the anode while reduction of oxygen takes place at the cathode (Fig. 2). Particularly, electroactive bacteria on the anode oxidize the organics in the wastewater producing electrons, protons, carbon dioxide and organic intermediates (Fig. 2) [34]. Protons, carbon dioxide and organics intermediates are released into the anodic solution while electrons flow through the external circuit generating positive electrical current [34]. Several oxidants were introduced for the cathodic reaction [73] but oxygen was most effective due to the natural availability (and therefore does not need to be supplied or refilled), low cost, and high electrochemical reduction potential. At the cathode, the oxygen reduction reaction (ORR) pathway can involve 2e^−^, 2e−x2e^−^ or 4e^−^ [74], [75], [76], [77], [78], [79] with final 4e^−^ product H_2_O or OH^−^ depending on the catalyst and the acidic or alkaline environment (Fig. 2) [76], [77]. When the MDC electrode reactions take place, the ions (Na^+^ and Cl^−^) move through the selective membranes to maintain electroneutrality. Na^+^ ions migrate through the cation exchange membrane (CEM) that allows positive ion transport from the desalination chamber to the cathode chamber (Fig. 2). Similarly, Cl^−^ ions move through the anion exchange membrane (AEM) from the desalination chamber to the anode chamber (Fig. 2). The transport of ions through the selective membrane is regulated by the Fick’s law in which the driving force is the gradient of concentration between the desalination cell and the anode and cathode chamber.

Consequently, ions move from the desalination chamber (more concentrated) to the anode and cathode chamber (less concentrated) via diffusion through the selective membranes. Osmosis also takes place with transport of water from the anode and cathode chamber (low concentration of ions) to the desalination chamber (high concentration of ions). A third process is named migration in which ions are electrostatically attracted by the self-polarized electrodes with the anode negatively charged and the cathode positively charged attracting cations and anions, respectively.

Redox reactions at both anode and cathode cause a potential difference between the two electrodes. At the anode, the bacteria that colonize the electrode consume the oxygen and create an anaerobic zone in which the potential is strongly pushed towards negative values. In parallel, the air-breathing configuration allowed oxygen presence at the cathode and consequently the potential was kept at high potentials. The anode and cathode surfaces are then self-polarized with the anode being the negative electrode and the cathode the positive electrode (Fig. 1a). The charge of the electrode is balanced by counter ions of dissolved ionic species into the solution (Fig. 1a). The anode (negative electrode) will attract positive ions and the cathode (positive electrode) will attract negative ions from the solution. The ions into the electrolyte migrate towards the oppositely charged electrode forming an electrochemical double layer at each of the electrode (Fig. 1a).

High surface area carbonaceous electrodes feature high-double layer capacitance and high charge storage capability, which in turn is balanced by a large amount of counter ions from the electrolyte. The electrodes store ions from the electrolyte similar to an electrochemical double layer capacitor (EDLC) (Fig. 1a). The same concept was previously exploited to demonstrate a supercapacitive microbial fuel cell [68], [69], [70], [71], [72]. Here, the concept is applied to a MDC device to improve both power/current generated and desalination efficacy. In fact, the negative and positive electrodes can be then discharged by fast and reversible electrostatic processes in which ions are released into the bulk electrolyte solution (Fig. 1a). The energy electrostatically stored can be delivered by short galvanostatic discharge pulses (GLV) generating high power output with no addition of external power. After the discharge (during rest), the electrodes restore their potential equilibrium, are polarized back, and the electrochemical double layers are formed again. Under those conditions, the electrodes work like the components of a self-powered internal supercapacitor and can be discharged/self-recharged theoretically infinite number of cycles. The position of the membranes in the supercapacitive microbial desalination cell (SC-MDC) are reversed compared to a traditional MDC (compare Fig. 1, Fig. 2) to facilitate the ions migration from the desalination chamber to the anode and cathode chamber by electrostatic attraction of negative and positive electrode respectively. In this configuration, migration of ions is enhanced by the self-polarization of the electrodes.

## 4 Results and discussion

### 4.1 Single galvanostatic discharge analysis of SC-MDC and SC-MDC-AdE

**Fig. 3:**
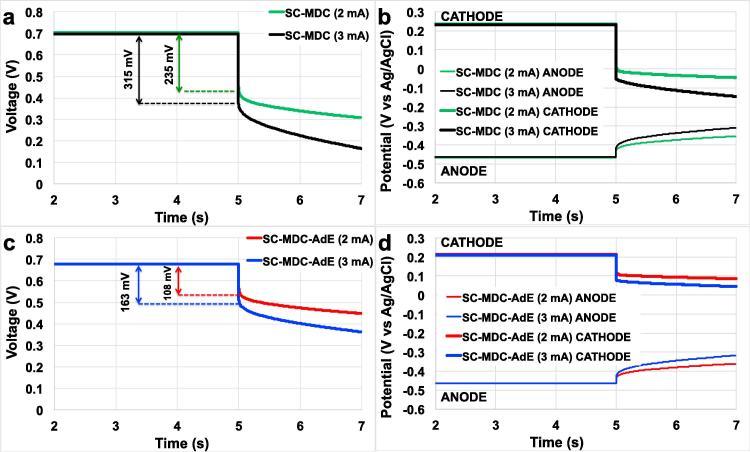
Cell voltage and electrode potential profiles (ANODE and CATHODE) of SC-MDC (a and b) and SC-MDC-AdE (c and d) under 5s rest and 2s pulses at i_pulse_ of 2 mA (2.9 A m^−2^) and 3 mA (4.3 A m^−2^).

Galvanostatic (GLV) discharges were carried out at pulse current (i_pulse_) of 2 mA (2.9 Am ^−2^) and 3 mA (4.3 A m^−2^) for a pulse time (t_pulse_) of 2 s for SC-MDC. Cell voltage (Fig. 3a) and electrode potential (anode and cathode) (Fig. 3b) discharge profiles of the SC-MDC are shown. SC-MDC had a ohmic drop (ΔV_ohmic_) of 235 mV and 315 mV at i_pulse_ of 2 mA and 3 mA respectively (Fig. 3a). Those values correspond to an equivalent series resistance (cell ESR) of 110 Ω. The main source of those losses was attributed to the cathode with a ΔV_ohmic,cathode_ of 201 mV and 292 mV at i_pulse_ of 2 mA and 3 mA, respectively (Fig. 3b). Those losses correspond to a cathodic ohmic resistance (R_C_) of roughly 100 Ω. Therefore, the contribution of the cathode on the overall cell ESR represents roughly for 90%.

Consequently, the brush anode had an anodic ohmic resistance (R_A_) of around 10 Ω that was approximately 10% of the R_C_ and also weighted only 10% on the cell ESR. In order to overcome the cathodic ohmic losses, an additional electrode (AdE) based on a small high surface area carbon brush coated with high surface area AC was inserted in the cathodic chamber. The titanium core in which the carbon fibers of the brush were twisted guaranteed low electrode ohmic losses. The activated carbon coated on the fibers instead assured high capacitance. The AdE was short circuited with the cathode in order to acquire the same potential of the cathode. In this case, the anode worked as negative electrode and the AdE worked as positive electrode of the internal supercapacitor. A significant decrease of ohmic drop (ΔV_ohmic_) was achieved by the addition of the additional electrode (AdE). Cell ESR values were reduced by about half by the addition of the AdE, in fact SC-MDC-AdE had ΔV_ohmic_ of 110 mV and 160 mV at i_pulse_ of 2 mA and 3 mA respectively (Fig. 3c). Correspondingly, the calculated cell ESR decreased from 110 Ω (SC-MDC) to 55 Ω (SC-MDC-AdE). The electrode profiles (anode and cathode) confirmed that the AdE decreased substantially the cathode ohmic losses without affecting anode performances (Fig. 3d). The contribution of the cathode was, however, still significant and was quantified to 40 Ω still representing 80% of the overall cell ESR (Fig. 3d).

The overall capacitance quantified over a t_pulse_ of 2 s also increased due to the AdE with measured values of 13 mF for SC-MDC and 17.5 mF for SC-MDC-AdE. Cathode capacitance doubled from 22 mF (SC-MDC) to 44 mF (SC-MDC-AdE); the anode capacitance remained stable at 23 mF.

### 4.2 Power curves of SC-MDC and SC-MDC-AdE

**Fig. 4:**
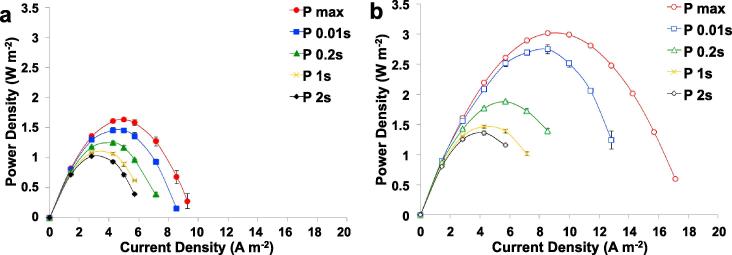
P_max_ and P_pulse_ for t_pulse_ of 2s (P 2s), 1s (P 1s), 0.2s (P 0.2s) and 0.01s (P 0.01s) for SC-MDC (a) and SC-MDC-AdE (b).

The SC-MDC and SC-MDC-AdE were galvanostatically discharged at different i_pulse_ for different t_pulse_. The trends of maximum power (P_max_) and the power for a certain pulse (P_pulse_) versus the current for t_pulse_ of 2s, 1s, 0.2s and 0.01s are shown in Fig. 4.

P_max_ was calculated at different current densities by taking the open circuit voltage (V_max_) of 0.67 V and cell ESR of ≈110 Ω for SC-MDC (Fig. 4a) and ≈55 Ω for SC-MDC (Fig. 4b). The highest P_max_ for SC-MDC was 1.63 ± 0.04 W m^−2^ (1.14 ± 0.03 mW) at 5 A m^−2^ (3.5 mA). P_max_ increased by roughly 90% with SC-MDC-AdE achieving 3.01 ± 0.01 W m^−2^ (2.11 ± 0.01 mW) at i_pulse_ of 8.5 A m^−2^ (6 mA). Those values are much higher than any power reported for MDC in literature [47], [48], [49], [58].

An increase of t_pulse_ corresponded to a reduction in P_pulse_. P_pulse_ for SC-MDC had the highest values of 1.02 ± 0.02 W m^−2^ (0.72 ± 0.01 mW) at i_pulse_ of 2.85 A m^−2^ (2 mA) for t_pulse_ of 2s, 1.08 ± 0.01 W ^−2^ (0.76 ± 0.01 mW) at i_pulse_ of 2.85 A m^−2^ (2 mA) for t_pulse_ of 1s, 1.25 ± 0.03 W m^−2^ (0.87 ± 0.02 mW) at i_pulse_ of 4.28 A m^−2^ (3 mA) for t_pulse_ of 0.2s and 1.46 ± 0.04 W m^−2^ (1.02 ± 0.03 mW) at i_pulse_ of 4.28 A m^−2^ (3 mA) for t_pulse_ of 0.01 s. SC-MDC-AdE displayed P_pulse_ that are 50–80% higher than SC-MDC’s. Particularly, P_pulse_ peak for t_pulse_ of 2 s was 1.36 ± 0.04 W m^−2^ (0.95 ± 0.03 mW) at i_pulse_ of 4.28 A m^−2^ (3 mA), for t_pulse_ of 1s was 1.46 ± 0.03 W m^−2^ (1.02 ± 0.02 mW) at i_pulse_ of 4.28 A m^−2^ (3 mA), for t_pulse_ of 0.2s was 1.88 ± 0.01 W m^−2^ (1.31 ± 0.01 mW) at i_pulse_ of 5.7 A m^−2^ (4 mA) and for t_pulse_ of 0.01s was 2.74 ± 0.07 W m^−2^ (1.92 ± 0.05 mW) at i_pulse_ of 8.6 A m^−2^ (6 mA).

### 4.3 Durability tests of SC-MDC and SC-MDC-AdE with an NaCl solution

**Fig. 5:**
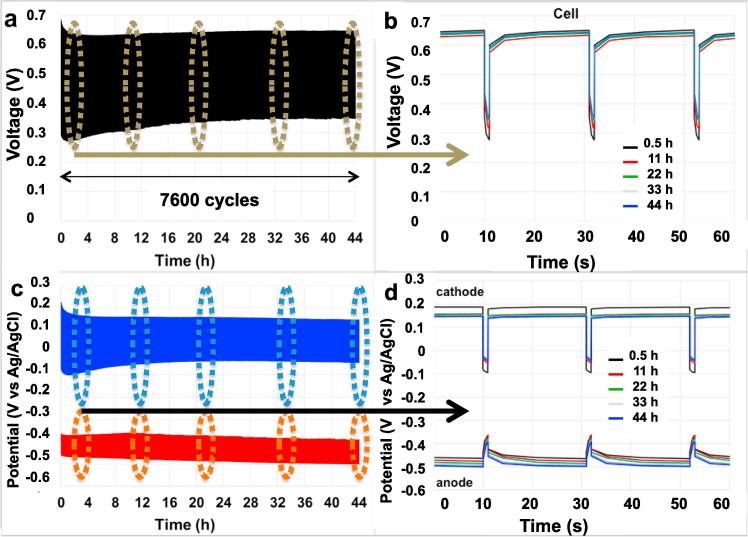
GLV discharge/self-recharge cycles of the SC-MDC during 44 h and magnification of the cycles after 0.5 h, 11 h, 22 h, 33 h and 44 h: cell voltage (a) and (b) and electrode potentials (c) and (d).

**Fig. 6:**
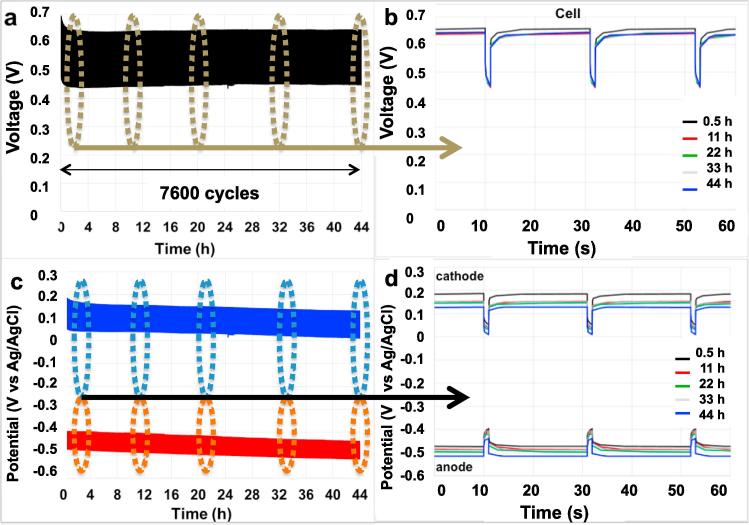
GLV discharge/self-recharge cycles of the SC-MDC-AdE during 44 h and magnification of the cycles after 0.5 h, 11 h, 22 h, 33 h and 44 h: cell voltage (a) and (b) and electrode potentials (c) and (d).

The SC-MDC and SC-MDC-AdE systems were tested for 44 h in batch mode continuously (Figs. 5and 6). 7600 discharge/self-recharge cycles were run for SC-MDC (Fig. 5a) and SC-MDC-AdE (Fig. 6a) at i_pulse_ of 2.9 A m^−2^ with t_pulse_ of 1s. A rest of 20s was required to restore the initial cell voltage and recharge the internal EDLC.

The V_max,OC_ of SC-MDC and of SC-MDC-AdE started from roughly 700 mV and after 30 min lost already 50 mV stabilizing at ≈650 mV (Figs. 5a and 6a). This loss was due to the cathode potential that was initially ≈225 mV (vs Ag/AgCl) and then stabilized at ≈185mV (vs Ag/AgCl) (Figs. 5c and 6c).

Cell voltage of the SC-MDC and SC-MDC-AdE after 0.5, 11, 22, 33 and 44 h are shown in Figs. 5b and 6b. Electrode potentials of the SC-MDC and SC-MDC-AdE after 0.5, 11, 22, 33 and 44 h are shown in Figs. 5d and 6d. Data related with open circuit voltage (OCV), open circuit potential (OCP), cell ESR, R_A_, R_C_, C_cell_, C_C_, C_A_ concerning SC-MDC and SC-MDC-AdE during the 44 h are shown in Tables 1 and 2 respectively.

**Table 1:** Values of maximum cell voltage (V_max,oc_) with the corresponding values of cathode (V^+^_, oc_) and anode potentials (V^−^_, oc_) in rest, cell ESR and Capacitance (C_cell_) of SC-MDC. Anode and Cathode resistances (R_A_, R_C_) and capacitances (C_A_, C_C_) estimated by the analysis of the single electrode potential profiles are also reported.

	SC-MDC		
	V^−^_, oc_	V^+^_, oc_	V_max, oc_
	(mV vs Ag/AgCl)	(mV vs Ag/AgCl)	(mV)
h	Anode	Cathode	Cell
0.5	−465 ± 14	187 ± 10	652 ± 11
11	−478 ± 12	158 ± 6	636 ± 8
22	−485 ± 8	157 ± 5	642 ± 5
33	−493 ± 4	151 ± 2	644 ± 2
44	−500 ± 4	147 ± 3	647 ± 2

	R_A_ (Ω)	R_C_ (Ω)	ESR (Ω)
h	Anode	Cathode	Cell

0.5	8 ± 1	102 ± 2	110 ± 2
11	11 ± 1	82 ± 2	93 ± 2
22	12 ± 1	75 ± 2	87 ± 2
33	12 ± 1	73 ± 2	85 ± 1
44	12 ± 1	73 ± 2	85 ± 2

	C_A_ (mF)	C_C_ (mF)	C_TOT_ (mF)
h	Anode	Cathode	Cell

0.5	26.6 ± 2.0	25.8 ± 2.8	13.1 ± 1.5
11	22.0 ± 1.1	40.1 ± 1.3	14.2 ± 0.7
22	23.6 ± 1.2	45.4 ± 0.4	15.5 ± 0.6
33	23.6 ± 1.0	46.8 ± 0.5	15.7 ± 0.4
44	24.2 ± 1.0	46.4 ± 0.2	15.9 ± 0.3

**Table 2:** Values of maximum cell voltage (V_max,oc_) with the corresponding values of cathode (V^+^_, oc_) and anode potentials (V^−^_, oc_) in rest, cell ESR and Capacitance (C_cell_) of SC-MDC-AdE. Anode and Cathode resistances (R_A_, R_C_) and capacitances (C_A_, C_C_) estimated by the analysis of the single electrode potential profiles are also reported.

	SC-MDC-AdE		
	V_−, oc_	V_+,oc_	V_max, oc_
	(mV vs Ag/AgCl)	(mV vs Ag/AgCl)	(mV)
h	Anode	Cathode	Cell
0.5	−471 ± 10	185 ± 9	656 ± 8
11	−484 ± 11	153 ± 6	637 ± 8
22	−497 ± 7	146 ± 6	643 ± 6
33	−498 ± 7	149 ± 4	647 ± 4
44	−512 ± 4	130 ± 2	642 ± 3

	R_A_ (Ω)	R_C_ (Ω)	ESR (Ω)
h	Anode	Cathode	Cell

0.5	11 ± 2	39 ± 2	50 ± 2
11	12 ± 1	39 ± 1	51 ± 1
22	12 ± 1	39 ± 1	51 ± 1
33	12 ± 1	39 ± 2	51 ± 2
44	12 ± 1	39 ± 1	51 ± 1

	C_A_ (mF)	C_C_ (mF)	C_TOT_ (mF)
h	Anode	Cathode	Cell

0.5	29 ± 2	36.0 ± 1.1	16.1 ± 1.0
11	29 ± 1	49.2 ± 0.4	18.2 ± 0.5
22	29 ± 1	55.3 ± 1.0	19.0 ± 0.7
33	30 ± 1	57.1 ± 0.3	19.7 ± 0.4
44	29 ± 1	55.4 ± 0.6	19.0 ± 0.6

The cell ESR of SC-MDC decreased from an initial value of 110 ± 2 Ω to 85 ± 2 Ω after 44 h (Table 1). This was due to a decrease in the R_C_ from 102 ± 2 Ω to 73 ± 2 Ω. The R_A_ remained stable between 8 ± 1 and 12 ± 1 Ω. Interestingly, the cathode is responsible for roughly 80–90% of the overall ohmic resistance. The addition of the AdE halved the cell ESR to 50 ± 2 Ω that remained stable over time (Table 2). Also in this case, R_C_ accounted for the majority of the total losses (39 ± 2 Ω), which remained stable over time (Table 2).

Interestingly, the capacitance (C_cell_) of both SC-MDC and SC-MDC-AdE increased its values upon cycling (Table 1, Table 2). C_A_ remained stable and was measured to be 22–30 mF independently from the presence of absence of the AdE (Table 1, Table 2). C_C_ increased significantly, almost doubling its initial value. In SC-MDC, C_C_ increased from 25.8 ± 2.8 mF (0 h) to 45.4 ± 0.2 mF (22 h) and then stabilized until 44h. The increase of C_C_ in SC-MDC increased also the C_cell_ that moved from 13.1 ± 1.5 mF (0 h) to 15.9 ± 0.3 (44 h). A similar trend was observed for C_C_ in SC-MDC-AdE. C_C_ increased from 36.0 ± 1.1 mF to 55.4 ± 0.6 mF within the 44 h and C_cell_ varied from 16.1 ± 1.0 mF to 19.0 ± 0.6 mF.

It is important to note that the additional electrode in the AdE system substantially decreased the R_C_ and increased the C_C_ and consequently has a beneficial effect on the overall cell performance. The change of resistance and capacitance of the cathode and, consequently, of the overall cell resistance and capacitance were probably due to the change of ionic composition and concentration of the cathode chamber that is discussed in the next section (Fig. 6).

### 4.4 Variation of solution parameters

**Fig. 7:**
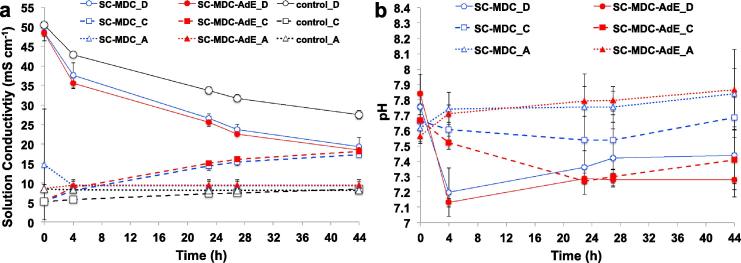
Solution conductivity (a) and pH (b) trend in the cathode, desalination and anode chambers of SC-MDC and SC-MDC-AdE during 44 h test. The labels D, C and A indicate the desalination, cathode and anode chambers, respectively. Red color represents SC-MDC-AdE, blue color represent SC-MDC and black color is representative for the control test. (For interpretation of the references to colour in this figure legend, the reader is referred to the web version of this article.)

The solution conductivity and pH of the three chambers were monitored during the 44 h-GLV pulse sequence described in the previous section. Conductivity in the DC decreased significantly in the first 24 h and then the desalination rate slowed down due to a lower ion gradient through the membranes (Fig. 7a). The solution conductivity in the DC measured in those experiments was 19.2 ± 2.3 mS cm^−1^ for SC-MDC and 18.3 ± 1.0 mS cm^−1^ for SC-MDC-AdE after 44 h. The decrease in salinity from the starting value was 45–47% after 23 h and 60–62% after 44 h. The solution conductivity of the anode chamber remained approximately constant during the experiments (Fig. 7a). After 44 h, the solution conductivity of the cathode chamber increased up to 17.2 ± 0.5 mS cm^−1^ and 18.1 ± 0.3 mS cm^−1^ in SC-MDC and SC-MDC-AdE respectively. It is interesting to note that the cathode chamber and desalination chamber ended at nearly the same conductivity at the end of 44 h indicating that the equilibrium was reached and further decrease in desalination was not possible. The fact that the solution conductivity in the cathode chamber increased can explain the decrease of R_C_ and increase of C_C_ measured over time (Fig. 6 and Table 1, Table 2).

A control experiment was run with the three chambers filled with the different solutions and solution conductivity was monitored over 44 h. In this latter case, anode and cathode electrodes were not inserted in the chamber in order to estimate the contribution due to the migration process. Interestingly, the conductivity of the desalination chamber decreased from an initial value of 50.5 mS cm^−1^ to 33.7 ± 1 mS cm^−1^ after 23 h and 27.5 ± 1.1 mS cm^−1^ after 44 h. The conductivity of the cathode chamber solution increased 5.15 mS cm^−1^ to 8.37 ± 0.3 mS cm^−1^. At last, the conductivity of the anode chamber did not vary much remaining constantly around 8.2–8.3 mS cm^−1^. The solution conductivity in the DC decreased much less compared to the SC-MDC indicating that in the latter system not just diffusion and osmosis are taking place, but also migration is contributing to ion transport.

Interestingly, the pH (Fig. 7b) did not vary dramatically in any chamber during the experiments, remaining between 7.0 and 8.1 in all chambers. Supercapacitive MDC works just electrostatically. Since the process is just attraction/rejection (adsorption/desorption) of charges, the production of reaction products that can alter the pH such H^+^ or OH^−^, as shown in Fig. 1, is practically absent. Significant increases in pH in the solutions were instead noticed in MDCs with pH values up to 10 [50], [59], [80]. High pH is not desired because of the possibility of salt precipitation on the membrane and consequent membrane fouling.

The COD was also measured and the initial COD was measured at 2365 ± 45 mg L^−1^. After 44 h the COD was 138 ± 10 mg L^−1^ for SC-MDC and 126 ± 9 mg L^−1^ for SC-MDC-AdE corresponding to a decrease in organics content of roughly 94%. Since the system was working in pulse mode as an internal supercapacitor (electrostatically), the degradation of organics might be due to fermentation processes different than electroactive biofilm degradation.

### 4.5 Durability tests of SC-MDC and SC-MDC-AdE with real seawater

Durability tests with 15,100 discharge-self-recharge tests (88 h) were run for SC-MDC and SC-MDC-AdE with the DC containing real seawater collected from the Pacific Ocean. The primary purpose of this test was to utilize real seawater rather than the synthetic one in order to simulate conditions as close as possible to the real application. The longer duration of this test was not preselected, but instead was the maximum possible time before the conductivity between the chambers was nearing equilibrium. This was due to the osmosis and evaporation processes taking place into the system. In fact, in parallel to diffusion and migration phenomena that are related to ions transport, water osmosis was occurring into the system with water moving from anode and cathode chamber to the central desalination chamber and actually overflowing from the outlet. The latter transport phenomena lead to a decrease in the liquid level on both anode and cathode chamber. Lower solution level on the anode might be undesirable because it could expose the anaerobic anode to the atmosphere leading to a decrease in performance. In parallel, also the decrease in the solution level into the cathode chamber could be unwanted since the cathode geometric area would not be completely in contact with the solution and therefore performances would be penalized. In respect to the cathode chamber, evaporation is also taking place due to the air-breathing cathode and therefore the solution level is subject to a much faster decrease.

**Fig. 8:**
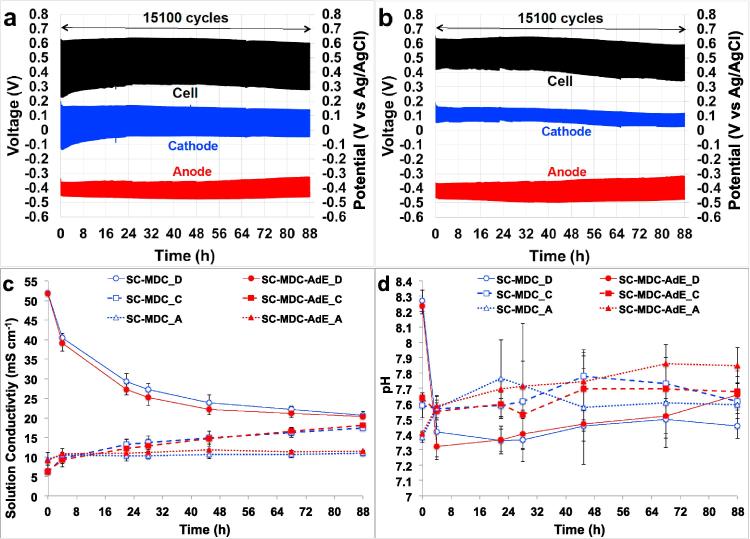
15,100 GLV discharge/self-recharge cycles of the SC-MDC (a) and SC-MDC-AdE (b) with DC having real seawater at i_pulse_ of 2.9 A m^−2^ for 88 h. Solution conductivity (c) and pH (d) trend in the cathode, desalination and anode chamber during the experimentation. Red color represents SC-MDC-AdE, blue color represents SC-MDC. (For interpretation of the references to colour in this figure legend, the reader is referred to the web version of this article.)

Performance was similar to that of the cells run with the NaCl solution (Fig. 8a). Also in this case, the AdE decreased the ohmic losses and consequently performances were increased significantly (Fig. 8b). Thick black lines indicate the 15,100 discharge/self-recharge cycles of the overall system (Fig. 8a and b), thick blue lines blue indicate the 15,100 discharge/self-recharge cycles of the cathode (Fig. 8a and b) and thick red lines indicate the 15,100 discharge/self-recharge cycles of the anode (Fig. 8a and b).

Cell ESR with SC-MDC-AdE was identified as 60 Ω at time 0 h while for SC-MDC, Cell ESR was 90% higher (112 Ω). Similarly, it can be noticed a decrease in the R_C_ over time in the SC-MDC (Fig. 8a). R_C_ remained instead constant for SC-MFDC-AdE (Fig. 8b).

Despite similar current/power pulses performances compared to the NaCl solution (30 g L^−1^ NaCl), quite different desalination rate was measured (Fig. 8c). In fact, solution conductivity inside the DC and inside the cathode chamber did not change as fast as with the NaCl solution containing just NaCl and this was probably due to the more complex composition of seawater compared to the NaCl solution. After 45 h operations, the solution conductivity of the DC was 23.85 ± 2.04 mS cm^−1^ for SC-MDC and 22.12 ± 1.39 mS cm^−1^ for SC-MDC-AdE (Fig. 8c). At the same time, the solution conductivity of the cathode chamber was 14.87 ± 1.67 mS cm^−1^ for SC-MDC and 14.61 ± 1.92 mS cm^−1^ for SC-MDC-AdE (Fig. 8c). This indicates that still after 44 h operations, a reduced but existing driving force was present between DC and the cathode chamber. After 88 h, the difference in solution conductivity between DC and cathode chamber was lower than 2 mS cm^−1^ indicating a very low salinity difference.

The initial pH of the seawater was slightly alkaline (8.25 ± 0.15) but during the experimentation the pH moved within the range of 7 and 8. The pH of anodic and cathodic chamber did not change also in this case, remaining in the circumneutral zone being suitable for water reuse (Fig. 8d). In this case, COD was also measured with initial value of 2220 ± 56 mg L^−1^ and a final value of 112 ± 12 mg L^−1^ for SC-MDC and 97 ± 33 mg L^−1^ for SC-MDC-AdE with a decrease of over 90%.

### 4.6 Comparison with existing literature

The existing literature on MDC is very diverse due to the different operating conditions utilized that affect the overall performances such as: (i) MDC design; (ii) working temperature; (iii) anion and cation exchange membrane; (iv) buffer and electrolytes; and (v) final electron acceptor such as oxygen or potassium ferricyanide [47], [49], [59]. Using the same operating conditions but working in standard MDC mode, the power generation obtained was 0.4–0.5 W m^−2^ that is actually roughly three times lower compared to SC-MDC and six times lower compared to SC-MDC-AdE [50]. Reduction in salinity content was also slight lower indicating the advantage of operating in supercapacitive mode [50]. Electrochemical performances can also be increased using potassium ferricyanide [47], [49], [59], using osmotic membranes [55], [81], [82] and certainly increasing the operating temperature to enhance the anode kinetics [83], [84]. Power output can also be increased by reducing the space between the anion and cation exchange membrane with a desalination chamber volume of 3 mL [35]. Also the increase in phosphate buffer molarity as electrolyte from 25 mM to 50 mM was reported to increase the power density from ≈0.8 W m^−2^ to 1.0 W m^−2^ [80]. At last, also the MFC stacking lead to an increase in power generation measured in roughly 1.2 W m^−2^ [85].

The discharge/self-recharge operation mode here presented, increased the power/current generated by 3–6 times. Moreover, from a practical point of view, it permits to improve the quality of current/power generated since the current/power produced is mainly of electrostatic nature and not much affected by the variability of the biochemical environment. Our work demonstrated that current/power was quite stable over the operational period and therefore of great interest for practical applications requiring pulse power. It was shown previously that intermittent operational mode is quite beneficial for energy harvesting in bioelectrochemical systems [86], [87].

Considering the reduction in salinity, generally MDC batch systems are capable of reducing the salinity content by roughly 40–65% as indicated by a recent review [47], [49], [59]. The percentage of reduction increased (up to 80%) with the reduction of the desalination volume and 100% can be achieved if the system operates in continuous flow [47], [49], [59]. The system here presented had a reduction of roughly 60–62% over 44 h batch cycle using 30 g L^−1^ NaCl and roughly 63% reduction in salinity content using real seawater (88 h batch mode). Those data indicate that the reduction in salinity content measured in these experiments is on the higher end of the salinity reduction identified within the MDC field for batch mode operations.

### 4.7 Outlook and directions

The exploitation of supercapacitive electrode features in microbial fuel cells (SC-MFC) was successfully shown recently with a significant boost in electrochemical performance output by at least one order of magnitude higher compared to existing literature. The same concept was here applied to a microbial desalination cell with the possibility of having a bioelectrochemical system having threefold functionality of: (i) generating electricity with higher output compared to existing MDCs by exploiting the supercapacitive properties of the electrodes; (ii) reducing significantly the salinity content in the desalination chamber using osmosis, diffusion and migration; and (iii) degrading organics/pollutants. For the first time, the integration of supercapacitive electrodes in a microbial desalination cell is presented.

Unlike MFCs and MDCs, the SC-MDC works in pulsed and intermittent mode and is capable of producing high power/current pulses roughly one order of magnitude higher compared with continuous MFC or MDC operation. The SC-MDC electrodes work as positive and negative electrodes of an internal supercapacitor. The utilization of a supercapacitive AdE further decreased the ohmic losses and increase in C_cell_. Power pulses in SC-MDC-AdE were almost double compared to SC-MDC and in both cases the maximum power achieved was several times higher compared to traditional MDCs as recently shown [50]. Cell capacitance is not competitive with that of conventional EDLCs due to the fact that the materials utilized are more inherited from electrodes used in bioelectrochemical systems rather than highly capacitive electrodes used for supercapacitors. Further improvements can be certainly achieved by a judicious decoration of the SC-MDC electrodes with tailored design, high specific capacitance materials.

Unlike electrodialysis or capacitive deionization, the potential of the positive and negative electrodes is not externally imposed but is self-set by the redox couple within the two electrodes. This allows the generation of a potential gradient with no addition of external power, making the system self-standing and self-charged. The potential of the self-polarized electrode can work as an additional driving force for the ions movement through the selective membranes in addition to osmosis and diffusion. The reduction in salinity is then assisted by the redox potential of the anodic and cathodic couple. In SC-MDC, the desalination rate is faster when the concentration gradient between desalination chamber and cathode chamber (first 24 h) is higher. This means that ion diffusion coupled with the osmosis plays an important role within the overall desalination process. The osmosis process can be detected with the increase in the water level into the desalination chamber that actually overflowed and the decrease in the liquid level in both anode and cathode chamber noticed during the experiments.

Interestingly, during the operation, the solution conductivity of the cathode chamber increased due to the ions migration and this allowed the decrease of the ohmic losses (cell ESR) and the capacitance of the cell (C_cell_). Contrary to the existing literature in which the pH of cathode chamber rises [50], [58], [78], the pH of the cathode chamber did not change at all, demonstrating that the process is mainly electrostatic and not faradic. The noticeable decrease of COD during operation at almost constant pH indicates that organics were probably degraded by fermentative bacteria.

The work presented here is a proof of concept that show that utilizing the red-ox reaction of a MDC is possible to charge two electrodes in positive and negative way and discharge them as a supercapacitor. Several problems and limitations were encountered during this experimentation. First, the desalination rate was quite low and in order to overcome this problem, further design should be developed having greater membrane surfaces exposed for enhancing the ion exchange, smaller desalination chamber and thinner membrane designed specifically for this system. Second, the reduction in salt content was about 60% with final concentration of NaCl of about 12–14 g L^−1^. At this salt concentration level, water is still not drinkable. Therefore, possible solutions at this problem should envision: (i) the operations in continuous flow operation mode that could possibly enhance the salt reduction to values below 0.3 g L^−1^ as required to make water drinkable [47], [49], [59]; (ii) utilization of different SC-MDCs hydraulically connected in series in which the outlet of the first SC-MDCs becomes the inlet of the second SC-MDCs and so on. The number of SC-MDCs should be designed to target a final salt concentration value of the desalination chamber of 0.3 g L^−1^. At last, recently MDCs were exploited as possible pretreatment before inserting salty water into reverse osmosis (RO) system for drinking water generation [48], [88]. The reduction of salt before RO could be beneficial for the RO operations by reducing osmotic pressure.

Third, the distance between AEM and cathode was important and consequently high ohmic resistances occurred and negatively affected the overall system electrochemical performances. Further investigations should address those limitations and provide a novel design that can enhance the desalination rate and further increase the production of electricity.

## 5 Conclusions

For the first time, the concept of SC-MDC is presented. SC-MDC allows simultaneous wastewater treatment, reduction in salinity content and high power/current pulses production. SC-MDC and SC-MDC-AdE produced high current/power pulses compared to traditional microbial desalination cells (MDC). Maximum powers achieved were 3.01 ± 0.01 W m^−2^ (2.11 ± 0.01 mW) (SC-MDC-AdE) and 1.63 ± 0.04 W m^−2^ (1.14 ± 0.03 mW) (SC-MDC) respectively. The reduction in salinity content was faster in the first 24 h when the concentration gradient between desalination chamber and cathode chamber was higher. The solution conductivity in the desalination chamber dropped by 45–47% after 23 h and by 60–62% after 44 h. The pH in all the chambers did not vary significantly and remained within a neutral range, demonstrating that the process is mainly electrostatic. Solution at neutral pHs should positively impact on cell cycle life because carbonate precipitation that may clog the air breathing cathode as well as membrane is avoided. Compared to electrodialysis and capacitive deionization, no external electricity/power is supplied but in the case of SC-MDC (or SC-MDC-AdE) electricity/power is produced.
